# Repetitive negative thinking is associated with subjective cognitive decline in older adults: a cross-sectional study

**DOI:** 10.1186/s12888-020-02884-7

**Published:** 2020-10-09

**Authors:** Marco Schlosser, Harriet Demnitz-King, Tim Whitfield, Miranka Wirth, Natalie L. Marchant

**Affiliations:** 1 Division of Psychiatry, Faculty of Brain Sciences, University College London, 6th Floor, Wing A, Maple House, 149 Tottenham Court Road, London, W1T 7NF UK; 2 Department of Psychology, Faculty of Psychology and Educational Sciences, University of Geneva, Geneva, Switzerland; 3 German Center for Neurodegenerative Diseases (DZNE) Dresden, Dresden, Germany

**Keywords:** Subjective cognitive decline, Repetitive negative thinking, Dementia, Psychological risk factors, Cognitive function, Memory complaints

## Abstract

**Background:**

In the absence of a cure or effective treatment for dementia, attention has shifted towards identifying risk factors for prevention. Subjective Cognitive Decline (SCD) describes self-perceived worsening of cognition despite unimpaired performance on neuropsychological tests. SCD has been associated with an increased dementia risk and steeper memory decline. Repetitive negative thinking (RNT) is a transdiagnostic process that manifests across several mental health disorders associated with increased vulnerability to dementia. RNT has thus been proposed as a candidate marker of risk for dementia and, relatedly, could contribute to the manifestation of SCD. We aimed to investigate the relationship between SCD and RNT alongside other proposed psychological risk/protective factors for dementia and cognitive decline.

**Methods:**

In a cross-sectional online survey, 491 older adults (mean = 64.9 years, *SD* = 4.2; 63.1% female) completed measures of RNT, personality traits, purpose in life, worry, rumination, and meditation practice. SCD was assessed continuously via self-perceived cognitive function (Neuro-QoL) and categorically via endorsement (yes/no) of memory complaints. Regression models, using a stepwise backwards elimination, were built to assess associations between SCD, demographics, and all risk/protective factors.

**Results:**

A total of 24.2% of participants reported memory complaints. In the final prediction models, RNT was the only psychological variable associated with lower self-perceived cognitive function and with a higher likelihood of memory complaints.

**Conclusions:**

This study empirically corroborates the theoretical relationship between SCD and RNT. Longitudinal studies are needed to establish whether RNT is a prodromal symptom or an independent risk factor, and whether RNT can be a promising construct for future research on SCD and dementia risk.

## Background

Subjective Cognitive Decline (SCD) is characterised by self-perceived worsening of cognition in the absence of impaired performance on objective cognitive tests [1, 2]. One explanation for this may be that the decline is too subtle to be detected by standard cognitive assessments. Indeed, clinical and epidemiological data suggest that SCD is associated with later memory decline and increased risk of dementia [3–5], especially in older adults who are concerned about their perceived cognitive decline [6]. However, the underlying aetiologies of SCD are likely heterogeneous. For instance, SCD has been associated with intermittent sleep disturbances and side-effects from medication [2]. In an effort to understand this heterogeneity, the SCD plus classification has been proposed to capture several characteristics that increase the risk of objective cognitive decline [1]. These characteristics include worries about SCD, seeking of medical help because of SCD, and an onset of SCD at age 60 or above [2].

Given the lack of effective interventions for curing or treating dementia, increasing importance has been given to dementia prevention [7]. This shift in research orientation has led to a growing interest in (i) refining risk factor profiles for dementia and early presentations of potential dementia (e.g., SCD, mild cognitive impairment) [1, 7], and (ii) developing preventative intervention approaches [8–12]. Alongside well-established cardiovascular risk factors such as hypertension, obesity, physical inactivity, and smoking [7, 13], psychological risk factors, such as depression and anxiety, are emerging as promising intervention targets [14–16]. An aspect frequently found in psychological risk factors associated with dementia risk is repetitive negative thinking (RNT) [17]. In this context, elucidating the role of RNT in the presentation of SCD is of particular interest.

RNT encompasses worry (i.e., negative thoughts about future-related content) and rumination (i.e., negative thoughts about past-related content). RNT is characterised by the intrusive, repetitive, and difficult-to-disengage-from nature of negative thoughts [18]. Increasing evidence supports RNT as a style of thinking that operates across different mental health disorders (i.e., a transdiagnostic process) [19–21] that have been associated with an increased vulnerability to dementia and cognitive decline [17]. For instance, RNT contributes to the development and maintenance of depression, anxiety, and post-traumatic stress disorder [21–23], all of which have been associated with cognitive decline and increased vulnerability to dementia (e.g., [16, 24, 25]). It is possible that the increased risk of dementia conferred by these mental health disorders may be driven by RNT. In a recent study of healthy, cognitively unimpaired adults aged 55 years and older, RNT – but not depression and anxiety – was associated with cognitive decline and neuroimaging biomarkers of Alzheimer’s disease (i.e., amyloid, tau) [26]. To further delineate the link between RNT and cognitive risk factor profiles and early manifestations of dementia trajectories, research is needed to examine whether RNT is elevated in individuals with SCD.

This study aimed to investigate the cross-sectional relationship between SCD – here, conceptualised as memory complaints – and RNT in older adults. Importantly, we aimed to examine if a potential link between SCD and RNT can be detected even when accounting for other psychological risk/protective factors for dementia, namely personality traits (neuroticism, conscientiousness, openness to experience, agreeableness), purpose in life, and meditation experience. Briefly, high neuroticism has been associated with increased dementia risk and cognitive impairment [27–29] and high conscientiousness with reduced dementia risk [30, 31]. Although findings on the relationship between the remaining three dimensions of the big five personality framework [32] and dementia risk have been more tentative, high openness to experience [27] and high agreeableness [28] might confer protective effects, whereas extraversion has not been independently linked to dementia risk [27]. Greater purpose in life has been related to higher perceived cognitive function, decreased cognitive decline, and reduced risk of mild cognitive impairment and Alzheimer’s disease [33–35]. A measure of meditation practice was included because recent theoretical frameworks [36, 37] and preliminary neuroimaging data [38] highlight the potential positive impact of meditation on healthy ageing. Lastly, measures of rumination and worry, both components of RNT, were included to assess the role of time-dependent negative thoughts (i.e., past- and future-directed) in the hypothesised relationship between SCD and RNT.

## Methods

### Procedures

This study used an anonymous online questionnaire to collect cross-sectional data. Participants were recruited via Prolific (www.prolific.co) [39]. Prolific is an on-demand online data collection service that offers a profiled and high-quality participant pool to which custom screeners can be applied. The custom screeners used for the present study were the inclusion criteria detailed below.

Individuals were informed that the study aimed to advance the understanding of the link between thinking patterns, subjective experiences, and well-being. Participants received 0.80€ for the survey, which took approximately 8 min to complete. All data were collected in July 2019.

### Participants

Three inclusion criteria were applied: firstly, individuals had to be at least 60 years old. Secondly, they had to indicate never having received a diagnosis of mild cognitive impairment or dementia. And thirdly, they had to report having a good understanding of the English language. A total of 523 individuals consented to participate and started the survey. Thirty-two participants did not complete the survey and were excluded, resulting in a total sample of 491 participants.

### Measures

Participants were asked to indicate demographic details (i.e., age, sex, education, country of residence).

SCD was assessed continuously via a measure of self-perceived cognitive function, namely, the Cognition Function– Short Form from the Neuro-QoL Item Bank v2.0 [40]. This 8-item measure assesses memory, attention, and reasoning difficulties using a 5-point Likert scale ranging from 1 (very often/cannot do) to 5 (never/none). Four items ask about the past *7 days* (e.g., “I had to read something several times to understand it”); and four items ask about how much difficulty participants *currently* have performing certain activities (e.g., “Planning for and keeping appointments that are not part of your weekly routine”). Total scale scores are computed by summing all item scores with higher scores reflecting higher levels of self-perceived cognitive function. The Neuro-Qol Item Bank v2.0 has displayed adequate psychometric properties [40]. In the present study, Cronbach’s alpha for the 8-item Cognition Function– Short Form was 0.90.

SCD was assessed categorically via questions about memory complaints because an impaired memory is the most frequently reported complaint in research on SCD (e.g., [1, 41, 42]) and because previous cross-sectional research has utilised similar approaches to classifying SCD (e.g., [43, 44]). Participants answered the following question: “Do you have memory complaints?”. If participants reported memory complaints, they were asked “Do these complaints worry you?”. If they reported being worried, they were also asked “Have you seen a medical professional for your memory complaints?”. In the context of this study, participants with memory complaints were classified as having SCD. In other words, the answer to the question “Do you have memory complaints?” was used as a binary outcome variable capturing SCD.

To measure RNT, the Perseverative Thinking Questionnaire (PTQ) was used [45]. The 15-item self-report measure uses a 5-point Likert scale ranging from 0 (never) to 4 (almost always) to capture how participants typically think about negative experiences or problems. Although RNT comprises both worry and rumination, the PTQ items are time-independent. Specifically, they do not specify whether negative thoughts are related to the past or the future. Total PTQ scores are the sum of all item scores. Higher scores reflect higher levels of repetitive negative thinking (possible range: 0 to 60). The PTQ has displayed good internal consistency and reliable factor structure across samples [45]. Cronbach’s alpha in the present study was 0.95.

Worry and rumination, aspects of RNT, were measured using the 8-item Penn State Worry Questionnaire [46] and the 10-item Rumination Response Scale [47], respectively. Both measures were included to assess aspects of future- versus past-directed negative thoughts that are not directly captured by the content-independent measure of RNT (i.e., the PTQ). The 8-item Penn State Worry Questionnaire uses a 5-point Likert scale ranging from 1 (not at all typical of me) to 5 (very typical of me). The 10-item Rumination Response Scale uses a 4-point Likert scale ranging from 1 (almost never) to 4 (almost always). Total worry and rumination scores are derived by summing all the respective item scores with higher scores indicating higher levels of worry and rumination, respectively. Both scales have displayed adequate psychometric properties [46, 47]. In the present study, Cronbach’s alpha for the 8-item Penn State Worry Questionnaire and the 10-item Rumination Response Scale was 0.96 and 0.84, respectively.

To measure purpose in life, the 6-item purpose in life subscale of the Well-being Scale was used [48], which has been used in previous research involving older adults [33]. This measure uses a 6-point Likert scale ranging from 1 (strongly disagree) to 6 (strongly agree) to capture levels of purpose in life. Total purpose in life scores are derived by summing all item scores, with higher scores indicative of higher levels of purpose in life (possible range: 6 to 36). The purpose in life subscale has displayed good psychometric properties [45]. In the present study, Cronbach’s alpha was 0.90.

To measure personality traits, the 10-item short version of the Big Five Inventory was used [49]. This measure uses a 5-point Likert scale ranging from 1 (disagree strongly) to 5 (agree strongly) to capture the personality traits of openness to experience, conscientiousness, extraversion, agreeableness, and neuroticism [32]. Each personality trait is captured by two items and total scores for each trait are computed by averaging the respective items scores. Higher subscale scores relate to higher levels of the respective trait. Using expert judgment and empirical item analyses, the abbreviated 10-item version was developed based on the standard 44-item Big Five Inventory [50]. Despite being substantially shorter, the 10-item version of the Big Five inventory has retained significant levels of reliability and validity and was recommended for the use in time-constrained research settings [46].

Participants reported whether they “practice meditation regularly (on average at least 2-3 times per week)” without including activities such as yoga, tai chi, qigong, and prayer. Those with a regular meditation practice were further asked to indicate for how long they have been regularly practicing meditation.

### Statistical analysis

We built the statistical models within a risk prediction framework. We developed a set of linear and logistic regression models with self-perceived cognitive function (i.e., SCD assessed continuously) and endorsement (yes/no) of memory complaints (i.e., SCD assessed categorically) as the continuous and binary outcome variable, respectively. We used a stepwise backwards elimination approach in which explanatory variables (i.e., all psychological risk/protective factors described above) were retained if they were associated with *p* < 0.01. This conservative threshold for inclusion was chosen to ensure model stability and reduce overfitting.

To that end, we first fitted univariable linear regression models to assess the unadjusted association between self-perceived cognitive function and all psychological risk/protective factors; and we fitted univariable logistic regression models to assess the unadjusted association between memory complaints (yes/no) and all psychological risk/protective factors. Second, we fitted multivariable linear and logistic regression models that included all explanatory variables that were associated with *p* < 0.01 in the univariable regression models. The final prediction models retained all explanatory variables that were associated with *p* < 0.01 in the multivariable models. Age, sex, and education were retained in all models based on their established association with cognitive decline and dementia. Sensitivity logistic regression analyses were conducted that only included participants who reported worries about their memory complaints (yes/no) because worries about memory complaints have been identified as a high-risk feature of the SCD-plus classification [1]. All analyses were conducted using Stata 13.0 [51].

Given the absence of previous studies on the relationship between RNT and self-perceived cognitive function or memory complaints in older adults at the time of data collection, no effect size estimates were available on which to base a formal power analysis for sample size determination. Given the novelty of this research question, we considered a large sample size of at least 450 participants to be a conservative estimate for informing hypothesis-generation in the context future of longitudinal studies.

## Results

The total sample of 491 participants had an age range of 60 to 86 years. Table 1 reports participant characteristics. Table 2 reports descriptive characteristics based on participants’ responses to questions about their memory.

**Table 1:** Demographics and descriptive characteristics (*N* = 491)

Variable	Mean (SD)^**a**^	Range
Age (years)	64.9 (4.2)	60 to 86
60 to 64 years – *n* (%)	278 (56.6%)	
65 to 69 years – *n* (%)	139 (28.3%)	
70 to 75 years – *n* (%)	61 (12.4%)	
75 years and older – *n* (%)	13 (2.6%)	
Sex – *n* (%)		
Female	310 (63.1%)	–
Education		
Total years spent in school and higher education	15.0 (3.2)	8 to 26
Current country of residence – *n* (%)		
United Kingdom	287 (58.5%)	–
United States	143 (29.1%)	–
Canada	10 (2.0%)	–
Ireland	3 (0.6%)	
France	3 (0.6%)	
Australia	2 (0.4%)	
Italy	2 (0.4%)	
Greece	2 (0.4%)	
Chile	2 (0.4%)	
Other (Austria, Estonia, Netherlands, Portugal, Spain, Sweden)	6 (1.2%)	–
Not reported	31 (6.3%)	
Subjective cognitive decline assessed continuously		
Self-perceived cognitive function (Neuro-QoL)	32.7 (5.8)	11 to 40
Subjective cognitive decline assessed categorically		
Memory complaints – *n* (%)	119 (24.2%)	–
Worried about memory complaints – *n* (%)	79 (16.1%)	–
Medical professional sought for memory complaints – *n* (%)	16 (3.3%)	–
Repetitive negative thinking^b^ (PTQ)	23.6 (10.7)	0 to 60
Rumination (RRS-10)	17.6 (5.3)	10 to 36
Worry (PSWQ-8)	19.5 (8.9)	8 to 40
Purpose in life (Ryff’s wellbeing subscale)	27.8 (6.6)	6 to 36
Openness to experience (BFI-10)	3.75 (0.94)	1 to 5
Conscientiousness (BFI-10)	3.95 (0.91)	1 to 5
Extraversion (BFI-10)	3.00 (1.15)	1 to 5
Agreeableness (BFI-10)	3.67 (0.85)	1 to 5
Neuroticism (BFI-10)	2.59 (1.12)	1 to 5
Regular meditation practice – *n* (%)	43 (8.8%)	–
Meditation experience (years)	13.2 (14.6)	1 month to 50 years

*Abbreviations*: *SD* standard deviation, *PTQ* Perseverative Thinking Questionnaire, *RRS-10* 10-item Rumination Response Scale, *PSWQ-8* 8-item Penn State Worry Questionnaire, *BFI-10* 10-item Big Five Inventory

^a^All statistics are mean (*SD*) unless otherwise specified

^b^*n* = 490

**Table 2:** Categorical classification of SCD via endorsement of memory complaints

Variables^a^	No memory complaints	Reported memory complaints (SCD)	Reported memory complaints and being worried about complaints	Reported memory complaints, being worried about complaints, and having sought medical professional
*n*	372	119	79	16
Age (years)^a^	64.8 (4.2)	65.2 (4.2)	65.0 (3.9)	65.3 (5.0)
Female – *n* (%)	238 (64.0%)	72 (60.5%)	51 (64.6%)	10 (62.5%)
Education (years)	15.0 (3.2)	15.1 (3.3)	15.1 (3.3)	16.1 (2.5)
Self-perceived cognitive function	33.7 (5.3)	29.6 (6.1)	29.0 (6.7)	28.1 (8.7)

*Abbreviations*: *SCD* subjective cognitive decline, *SD* standard deviation, *SCD* subjective cognitive decline

^a^All statistics are mean (*SD*) unless otherwise specified

Table 3 displays the results of the univariable regression analyses. In the univariable linear regression models, RNT, worry, rumination, purpose in life, and the personality traits conscientiousness, extraversion, agreeableness, and neuroticism were associated with self-perceived cognitive function, the continuous outcome variable capturing SCD. In the univariable logistic regression models, RNT, worry, purpose in life, and the personality traits conscientiousness and neuroticism were associated with memory complaints, the binary outcome variable capturing SCD.

**Table 3:** Unadjusted associations with SCD assessed continuously via self-perceived cognitive function and SCD assessed categorically via endorsement of memory complaints (*N* = 491)

	Self-perceived cognitive function (standardised)			Memory complaints (binary)		
	Univariable linear regression models			Univariable logistic regression models		
Explanatory variable	Coefficient^a^	95% CI	***p***-value	Odds ratio^b^	95% CI	***p***-value
Age (1 year)	0.01	−0.01 to 0.03	0.461	1.02	0.97 to 1.07	0.450
Female (vs male)	0.11	−0.07 to 0.29	0.236	0.86	0.56 to 1.32	0.494
Education (1 year)	0.02	−0.01 to 0.05	0.154	1.01	0.95 to 1.08	0.714
Repetitive negative thinking (1 *SD*)^c^	−0.52	− 0.59 to − 0.44	< 0.001	1.78	1.43 to 2.21	< 0.001
Rumination (1 *SD*)	−0.39	− 0.48 to − 0.31	< 0.001	1.24	1.01 to 1.52	0.036
Worry (1 *SD*)	−0.48	−0.56 to − 0.40	< 0.001	1.55	1.26 to 1.90	< 0.001
Purpose in life (1 *SD*)	0.42	0.34 to 0.50	< 0.001	0.66	0.56 to 0.84	< 0.001
Openness to experience (1 *SD*)	0.11	0.02 to 0.20	0.017	1.02	0.83 to 1.25	0.873
Conscientiousness (1 *SD*)	0.27	0.18 to 0.35	< 0.001	0.74	0.60 to 0.90	0.003
Extraversion (1 *SD*)	0.26	0.17 to 0.34	< 0.001	0.76	0.61 to 0.94	0.011
Agreeableness (1 *SD*)	0.21	0.12 to 0.29	< 0.001	0.78	0.63 to 0.96	0.017
Neuroticism (1 *SD*)	−0.41	−0.50 to −0.33	< 0.001	1.54	1.25 to 1.90	< 0.001
Regular meditation practice (vs no practice)	−0.22	−0.54 to 0.09	0.159	1.08	0.53 to 2.22	0.829

*Abbreviations*: *SCD* subjective cognitive decline, *SD* standard deviation, *CI* confidence interval

^a^In the univariable linear regression models, the coefficient for the standardised continuous explanatory variables (indicated in parentheses by 1 *SD*) is equal to the Pearson’s correlation coefficient

^b^In the univariable logistic regression models, for binary explanatory variables (sex, regular meditation practice), the estimate describes the odds of memory complaints in one group relative to the reference category (indicated in parentheses). For continuous explanatory variables, the estimate reflects the expected increase in the odds of memory complaints for a one unit increase in the explanatory variable

^c^*n* = 490

Table 4 reports the results from the multivariable regression analyses. The final prediction model for self-perceived cognitive function retained age, sex, education, and RNT (standardised estimate = − 0.52; 95% CI: − 0.59 to − 0.44; *p* < 0.001); and the final prediction model for memory complaints (yes/no) retained age, sex, education, and RNT (odds ratio = 1.81; 95% CI: 1.46 to 2.26; *p* < 0.001). In other words, in the final linear and logistic regression models, higher levels of RNT were associated with worse self-perceived cognition and a higher likelihood of having reported memory complaints, respectively. Sensitivity logistic regression analyses that used worries about memory complaints as the binary outcome variable also resulted in a final prediction model that included age, sex, education, and RNT (odds ratio = 2.20; 95% CI: 1.70 to 2.86; *p* < 0.001) (see Additional file 1).

**Table 4:** Adjusted associations with SCD assessed continuously via self-perceived cognitive function and SCD assessed categorically via endorsement of memory complaints, and final prediction models (*n* = 490)

	Self-perceived cognitive function (standardised)			Memory complaints (binary)		
	**Adjusted models**^a^					
	**Multivariable linear regression model**			**Multivariable logistic regression model**		
**Explanatory variables**	**Coefficient**	**95% CI**	***p*-value**	**Odds ratio**^b^	**95% CI**	***p*-value**
Age (1 year)	− 0.002	−0.02 to 0.02	0.850	1.03	0.98 to 1.08	0.258
Female (vs male)	0.15	−0.01 to 0.31	0.063	0.85	0.54 to 1.34	0.477
Education (1 year)	0.01	−0.01 to 0.04	0.238	1.02	0.95 to 1.09	0.586
Repetitive negative thinking (1 *SD*)	−0.24	− 0.36 to − 0.12	< 0.001	1.64	1.16 to 2.31	0.005
Rumination (1 *SD*)	−0.11	−0.20 to − 0.01	0.026	–	–	–
Worry (1 *SD*)	−0.12	− 0.26 to 0.02	0.103	0.94	0.64 to 1.37	0.734
Purpose in life (1 *SD*)	0.12	0.02 to 0.21	0.017	0.96	0.74 to 1.25	0.768
Conscientiousness (1 *SD*)	0.10	0.02 to 0.18	0.011	0.84	0.67 to 1.06	0.146
Extraversion (1 *SD*)	0.06	−0.02 to 0.14	0.169	–	–	–
Agreeableness (1 *SD*)	0.03	−0.05 to 0.11	0.482	–	–	–
Neuroticism (1 *SD*)	−0.05	−0.16 to 0.07	0.418	1.18	0.86 to 1.62	0.306
	**Final prediction models**^c^					
	**Multivariable linear regression model**			**Multivariable logistic regression model**		
**Explanatory variables**	**Coefficient**	**95% CI**	***p*-value**	**Odds ratio**^b^	**95% CI**	***p*-value**
Age (1 year)	0.001	−0.02 to 0.02	0.931	1.03	0.98 to 1.08	0.256
Female (vs. male)	0.16	0.003 to 0.32	0.046	0.83	0.54 to 1.30	0.418
Education (1 year)	0.01	−0.01 to 0.04	0.252	1.02	0.96 to 1.09	0.502
Repetitive negative thinking (1 *SD*)	−0.52	− 0.59 to − 0.44	< 0.001	1.81	1.46 to 2.26	< 0.001

One participant had missing data on repetitive negative thinking and thus all models include 490 participants. Abbreviations: *SCD*, subjective cognitive decline; *SD*, standard deviation; CI, confidence interval

^a^The adjusted models include all explanatory variables with *p* < 0.01 in the univariable regression models. Age, sex, and education were retained based on their well-established association with dementia

^b^In the multivariable logistic regression model, for binary explanatory variables (sex), the estimate describes the odds of memory complaints in one group relative to the reference category (indicated in parentheses) when controlling for all other variables in the model. For continuous explanatory variables, the estimate reflects the expected increase in the odds of memory complaints for a one unit increase (indicated in parentheses) in the explanatory variable when controlling for all other variables in the model

^c^The final prediction models retained all predictors with *p* < 0.01 in the adjusted models. Age, sex, and education were retained based on their well-known association with dementia

## Discussion

This study investigated the relationship between RNT and SCD alongside a range of proposed psychological risk/protective factors for cognitive decline and dementia. Our results indicate that only RNT was associated with SCD. Specifically, higher levels of RNT were related to lower levels of self-perceived cognition and a higher likelihood of memory complaints. This association between RNT and SCD is particularly compelling because RNT has been theoretically and empirically linked with dementia risk [8, 26] and because no previous study had explicitly examined this relationship. SCD is a relatively newly defined clinical presentation with heterogeneous underlying aetiologies and requires more research to allow clearer predictions of its clinical trajectories. SCD has been associated with elevated anxiety and neuroticism, but we show that neither worry (a core symptom of anxiety), neuroticism, nor rumination (a core symptom of depression) add explanatory power to predict SCD symptoms over and above RNT. The relationship between RNT and SCD therefore appears not to be based on the time orientation of negative thoughts (worry being future-directed, rumination past-directed). In other words, our findings suggest that the transdiagnostic conceptualisation of RNT parsimoniously captures those facets of rumination and worry that are associated with SCD.

SCD-plus features – including worries about memory complaints – have been linked to an increased risk of objective cognitive decline. In line with our primary analyses, results from our sensitivity analyses, which only included participants who reported worries about memory complaints (i.e., excluded participants who reported memory complaints but who were not worried about these complaints), found that RNT was the only variable associated with this SCD-plus feature. It could be argued that because of the potential circularity between worries about memory complaints and RNT (i.e., a construct comprising worry), a relationship is to be expected. However, the fact that a separate gold standard measure of worry was not associated with worries about SCD challenges this circularity, and suggests that worry-independent facets of RNT also have utility for predicting SCD-plus.

Levels of reported purpose in life were associated with SCD in the univariable regression model. Previous research on older adults that used the same measure of purpose in life and a similar measure of self-perceived cognitive function also found a univariable relationship [33]. The association of purpose in life with SCD disappeared when adjusting for other psychological risk/protective factors, age, sex, and education. Similarly, conscientiousness only evidenced a relationship with SCD in the unadjusted model. There was no evidence that personality traits were associated with SCD when adjusting for age, sex, education, and other psychological factors.

The high prevalence of regular meditation practice observed in the present sample (point prevalence: 8.8%) is in line with recent prevalence estimates from the National Health Interview Survey (12-month prevalence: 13.4%) [52], a nationally representative and continuously fielded survey of the US population that indicates substantial increases in the use of meditation between 2012 and 2017. In the present sample, participants with a regular meditation practice were equally as likely to report lower self-perceived cognitive function or memory complaints than those without a regular meditation practice. Although some studies suggest that mindfulness meditation practice may positively affect selective and executive attention early on [53], it remains unclear whether short-term meditation practice can improve cognitive ability, in general, or reduce memory complaints, in particular [54]. Future research is needed that assesses participants’ meditation practice more comprehensively, including type of practice, duration, retreat experience, and intention to practice. Relatedly, recent theory and research discusses the potential benefits of meditation for (brain) health and cognition in ageing and for repetitive negative thinking in the context of a longer-term commitment to a regular meditation practice [36, 55, 56]. Shorter-term meditation-based interventions tend to focus primarily on affective outcomes such as anxiety and depressive symptoms [8], which have been shown to be reduced by relatively brief meditation retreats [57].

Several important limitations need to be acknowledged when interpreting our findings. Firstly, the cross-sectional nature of our data does not allow us to draw causal inferences about the relationship between self-perceived cognitive function, memory complaints, and the psychological risk/protective factors. Although our hypotheses and statistical approaches were informed by a theoretical framework that posits RNT as causally contributing to the manifestation of SCD and dementia, longitudinal studies are needed to understand this relationship. Secondly, SCD is heterogeneous. For some individuals, SCD may be associated with dementia (either as a risk factor or prodromal feature), for others SCD could be due to other causes. Given the available data we were unable to exclude SCD due to causes unrelated to dementia, therefore are limited in the inferences that can be made about the relationship between RNT and dementia risk. Future research that assesses more established risk factors for dementia (e.g., APOE genotype, cardiovascular function, depression, anxiety, hearing loss) [7] in addition to other potential causes of SCD (e.g., medication use, physical health) are needed to clarify the role of RNT in this risk profile. Thirdly, we exclusively relied on self-report data from an online recruitment platform; we did not obtain objective assessments of cognition and, therefore, cannot indicate whether participants showed a cognitively normal performance for their age. We were, however, able to refine the categorical classification of SCD based on participants’ worries about their memory complaints because those actively worried have been shown to be at higher risk of developing dementia [6], and the results remained unchanged. Lastly, generalisability of these findings may be limited due the present sample’s high levels of education.

In the context of dementia prevention research, the likelihood of developing effective intervention approaches is increased if the modifiability of candidate intervention targets has already been established. A particularly promising feature of RNT is its responsiveness to psychological interventions [19, 22], which distinguishes it from potentially more treatment-resistant psychological risk factors for dementia (e.g., personality traits). Future research is needed to investigate whether reducing levels of RNT is longitudinally associated with improved cognitive outcomes and lower incidence of SCD and dementia. Addressing RNT as an intervention target could also help promote well-being and improved mental health in older adults more broadly.

## Conclusion

In sum, this is the first study to investigate the relationship between RNT and SCD in older adults. Among a range of proposed psychological risk/protective factors for dementia, RNT emerged as the only predictor of SCD. Increasing our understanding of the link between RNT and SCD could potentially inform future dementia prevention strategies.

## Supplementary information

**Additional file 1: Table A.** Unadjusted associations with SCD-plus assessed categorically via endorsement of worries about memory complaints (*N* = 491). **Table B.** Adjusted associations with SCD-plus assessed categorically via endorsement of worries about memory complaints, and final prediction model (*n* = 490).

## Data Availability

All data and analysis scripts are available at the Open Science Framework: https://osf.io/ykz67/.

## Abbreviations

SCD: Subjective Cognitive Decline
RNT: Repetitive Negative Thinking
CI: Confidence Interval
SD: Standard Deviation

## References

[1] Jessen F, Amariglio RE, van Boxtel M, Breteler M, Ceccaldi M, Chételat G (2014). A conceptual framework for research on subjective cognitive decline in preclinical Alzheimer's disease. Alzheimers Dement.

[2] Jessen F, Amariglio RE, Buckley RF, van der Flier WM, Han Y, Molinuevo JL (2020). The characterisation of subjective cognitive decline. Lancet Neurol.

[3] Mitchell AJ, Beaumont H, Ferguson D, Yadegarfar M, Stubbs B (2014). Risk of dementia and mild cognitive impairment in older people with subjective memory complaints: meta-analysis. Acta Psychiatr Scand.

[4] Geerlings M, Jonker C, Bouter L, Ader H, Schmand B (1999). Association between memory complaints and incident Alzheimer's disease in elderly people with normal baseline cognition. Am J Psychiatry.

[5] Koppara A, Wagner M, Lange C, Ernst A, Wiese B, König H-H (2015). Cognitive performance before and after the onset of subjective cognitive decline in old age. Alzheimers Dement.

[6] Jessen F, Wiese B, Bachmann C, Eifflaender-Gorfer S, Haller F, Kölsch H (2010). Prediction of dementia by subjective memory impairment: effects of severity and temporal association with cognitive impairment. Arch Gen Psychiatry.

[7] Livingston G, Sommerlad A, Orgeta V, Costafreda SG, Huntley J, Ames D (2017). Dementia prevention, intervention, and care. Lancet.

[8] Marchant NL, Barnhofer T, Klimecki OM, Poisnel G, Lutz A, Arenaza-Urquijo E (2018). The SCD-well randomized controlled trial: effects of a mindfulness-based intervention versus health education on mental health in patients with subjective cognitive decline (SCD). Alzheimers Dement.

[9] Poisnel G, Arenaza-Urquijo E, Collette F, Klimecki OM, Marchant NL, Wirth M (2018). The Age-Well randomized controlled trial of the Medit-Ageing European project: Effect of meditation or foreign language training on brain and mental health in older adults. Alzheimers Dement.

[10] Ngandu T, Lehtisalo J, Solomon A, Levälahti E, Ahtiluoto S, Antikainen R (2015). A 2 year multidomain intervention of diet, exercise, cognitive training, and vascular risk monitoring versus control to prevent cognitive decline in at-risk elderly people (FINGER): a randomised controlled trial. Lancet.

[11] van Charante EPM, Richard E, Eurelings LS, van Dalen J-W, Ligthart SA, van Bussel EF (2016). Effectiveness of a 6-year multidomain vascular care intervention to prevent dementia (preDIVA): a cluster-randomised controlled trial. Lancet.

[12] Kivipelto M, Mangialasche F, Ngandu T. Lifestyle interventions to prevent cognitive impairment, dementia and Alzheimer disease. Nat Rev Neurol. 2018;14(11):653–66. 10.1038/s41582-018-0070-3 30291317

[13] Norton S, Matthews FE, Barnes DE, Yaffe K, Brayne C. Potential for primary prevention of Alzheimer's disease: an analysis of population-based data. Lancet Neurol. 2014;13(8):788–94. 10.1016/S1474-4422(14)70136-X 25030513

[14] Cherbuin N, Kim S, Anstey KJ (2015). Dementia risk estimates associated with measures of depression: a systematic review and meta-analysis. BMJ Open.

[15] Becker E, Rios CL, Lahmann C, Ruecker G, Bauer J, Boeker M (2018). Anxiety as a risk factor of Alzheimer's disease and vascular dementia. Br J Psychiatry.

[16] Gimson A, Schlosser M, Huntley JD, Marchant NL (2018). Support for midlife anxiety diagnosis as an independent risk factor for dementia: a systematic review. BMJ Open.

[17] Marchant NL, Howard RJ (2015). Cognitive debt and Alzheimer's disease. J Alzheimers Dis.

[18] Ehring T, Watkins ER (2008). Repetitive negative thinking as a transdiagnostic process. Int J Cogn Ther.

[19] Harvey A, Watkins E, Mansell W, Shafran R (2004). Cognitive behavioural processes across disorders: a transdiagnostic approach to research and treatment.

[20] Trick L, Watkins E, Windeatt S, Dickens C (2016). The association of perseverative negative thinking with depression, anxiety and emotional distress in people with long term conditions: a systematic review. J Psychosom Res.

[21] Spinhoven P, van Hemert AM, Penninx BW (2018). Repetitive negative thinking as a predictor of depression and anxiety: a longitudinal cohort study. J Affect Disord.

[22] Topper M, Emmelkamp PMG, Ehring T (2010). Improving prevention of depression and anxiety disorders: repetitive negative thinking as a promising target. Appl Prev Psychol.

[23] Ho FY, Chan CS, Tang KN (2016). Cognitive-behavioral therapy for sleep disturbances in treating posttraumatic stress disorder symptoms: a meta-analysis of randomized controlled trials. Clin Psychol Rev.

[24] Desmarais P, Weidman D, Wassef A, Bruneau MA, Friedland J, Bajsarowicz P, et al. The interplay between post-traumatic stress disorder and dementia: a systematic review. Am J Geriatr Psychiatr. 2019. 10.1016/j.jagp.2019.08.006 31488352

[25] Diniz BS, Butters MA, Albert SM, Dew MA, Reynolds CF (2013). Late-life depression and risk of vascular dementia and Alzheimer's disease: systematic review and meta-analysis of community-based cohort studies. Br J Psychiatry.

[26] Marchant NL, Lovland LR, Jones R, Pichet Binette A, Gonneaud J, Arenaza-Urquijo EM, Chételat G, Villeneuve S, PREVENT-AD Research Group. Repetitive negative thinking is associated with amyloid, tau, and cognitive decline. Alzheimers Dement. 2020;16:1054–64. 10.1002/alz.12116 32508019

[27] Low LF, Harrison F, Lackersteen SM (2013). Does personality affect risk for dementia? A systematic review and meta-analysis. Am J Geriatr Psychiatry.

[28] Terracciano A, Stephan Y, Luchetti M, Albanese E, Sutin AR (2017). Personality traits and risk of cognitive impairment and dementia. J Psychiatr Res.

[29] Johansson RL, Guo RX, Duberstein RP, Hällström RT, Waern RM, Östling RS (2014). Midlife personality and risk of Alzheimer disease and distress: a 38-year follow-up. Neurology..

[30] Sutin AR, Stephan Y, Terracciano A (2018). Facets of Conscientiousness and risk of dementia. Psychol Med.

[31] Kaup AR, Harmell AL, Yaffe K (2019). Conscientiousness is associated with lower risk of dementia among Black and white older adults. Neuroepidemiology..

[32] John OP, Naumann LP, Soto CJ (2008). Paradigm shift to the integrative big five trait taxonomy. Handb Pers.

[33] Bartrés-Faz D, Cattaneo G, Solana J, Tormos JM, Pascual-Leone A. Meaning in life: resilience beyond reserve. Alzheimers Res Ther. 2018;10(1):47. 10.1186/s13195-018-0381-z. 10.1186/s13195-018-0381-z PMC5968537 29793549

[34] Kim G, Shin S, Scicolone M, Parmelee P (2019). Purpose in life protects against cognitive decline among older adults. Am J Geriatr Psychiatry.

[35] Boyle PA, Buchman AS, Barnes LL, Bennett DA (2010). Effect of a purpose in life on risk of incident Alzheimer disease and mild cognitive impairment in community-dwelling older persons. Arch Gen Psychiatry.

[36] Chételat G, Lutz A, Arenaza-Urquijo E, Collette F, Klimecki O, Marchant N (2018). Why could meditation practice help promote mental health and well-being in aging?. Alzheimers Res Ther.

[37] Klimecki O, Marchant NL, Lutz A, Poisnel G, Chételat G, Collette F (2019). The impact of meditation on healthy ageing — the current state of knowledge and a roadmap to future directions. Curr Opin Psychol.

[38] Chételat G, Mézenge F, Tomadesso C, Landeau B, Arenaza-Urquijo E, Rauchs G (2017). Reduced age-associated brain changes in expert meditators: a multimodal neuroimaging pilot study. Sci Rep.

[39] Palan S, Schitter C (2018). Prolific. Ac—a subject pool for online experiments. J Behav Exp Financ.

[40] Cella D, Lai JS, Nowinski CJ, Victorson D, Peterman A, Miller D (2012). Neuro-QOL: brief measures of health-related quality of life for clinical research in neurology. Neurology..

[41] Mendonça MD, Alves L, Bugalho P (2016). From subjective cognitive complaints to dementia: who is at risk?: a systematic review. Am J Alzheimers Dis Other Dement.

[42] Burmester B, Leathem J, Merrick P (2016). Subjective cognitive complaints and objective cognitive function in aging: a systematic review and meta-analysis of recent cross-sectional findings. Neuropsychol Rev.

[43] Van Rooden S, van den Berg-Huysmans AA, Croll PH, Labadie G, Hayes JM, Viviano R, et al. Subjective cognitive decline is associated with greater white matter hyperintensity volume. J Alzheimers Dis. 2018;66(3):1283–94. https://content.iospress.com/articles/journal-of-alzheimers-disease/jad180285. 10.3233/JAD-180285 PMC6294575 30412485

[44] Flatt JD, Johnson JK, Karpiak SE, Seidel L, Larson B, Brennan-Ing M (2018). Correlates of subjective cognitive decline in lesbian, gay, bisexual, and transgender older adults. J Alzheimers Dis.

[45] Ehring T, Zetsche U, Weidacker K, Wahl K, Schönfeld S, Ehlers A (2011). The perseverative thinking questionnaire (PTQ): validation of a content-independent measure of repetitive negative thinking. J Behav Ther Exp Psychiatry.

[46] Hopko DR, Stanley MA, Reas DL, Loebach Wetherell J, Gayle Beck J, Novy DM (2003). Assessing worry in older adults: confirmatory factor analysis of the Penn State worry questionnaire and psychometric properties of an abbreviated model. Psychol Assess.

[47] Treynor W, Gonzalez R, Nolen-Hoeksema S (2003). Rumination reconsidered: a psychometric analysis. Cogn Ther Res.

[48] Ryff CD, Keyes CLM (1995). The structure of psychological well-being revisited. J Pers Soc Psychol.

[49] Rammstedt B, John OP (2007). Measuring personality in one minute or less: a 10-item short version of the big five inventory in English and German. J Res Pers.

[50] John OP, Donahue EM, Kentle RL. Big five inventory. J Pers Soc Psychol. 1991.

[51] StataCorp LP. Stata: release 13-statistical software: College Station; 2013.

[52] Clarke TC, Barnes PM, Black LI, Stussman BJ, Nahin RL. Use of yoga, meditation, and chiropractors among US adults aged 18 and over: US Department of Health and Human Services, Centers for Disease Control and Prevention, National Center for Health Statistics; 2018.

[53] Chiesa A, Calati R, Serretti A (2011). Does mindfulness training improve cognitive abilities? A systematic review of neuropsychological findings. Clin Psychol Rev.

[54] Lao S-A, Kissane D, Meadows G (2016). Cognitive effects of MBSR/MBCT: a systematic review of neuropsychological outcomes. Conscious Cogn.

[55] Lutz A, Klimecki OM, Collette F, Poisnel G, Arenaza-Urquijo E, Marchant NL, De La Sayette V, Rauchs G, Salmon E, Vuilleumier P, Frison E (2018). The age-well observational study on expert meditators in the Medit-ageing European project. Alzheimers Dement.

[56] Schlosser M, Jones R, Demnitz-King H, Marchant NL. Meditation experience is associated with lower levels of repetitive negative thinking: the key role of self-compassion. Curr Psychol. 2020.

[57] Khoury B, Knäuper B, Schlosser M, Carrière K, Chiesa A. Effectiveness of traditional meditation retreats: A systematic review and meta-analysis. J Psychosom Res. 2017;92:16–25. 10.1016/j.jpsychores.2016.11.006 27998508

