# Supplementary material for: Repetitive negative thinking is associated with subjective cognitive decline in older adults: a cross-sectional study
Source: BMC Psychiatry. 2020 Oct 9;20:500. doi: 10.1186/s12888-020-02884-7 (PMC7547434; doi:10.1186/s12888-020-02884-7)
Supplement: Supplementary file 1 — Additional file 1: Table A. Unadjusted associations with SCD-plus assessed categorically via endorsement of worries about memory complaints (N = 491). Table B. Adjusted associations with SCD-plus assessed categorically via endorsement of worries about memory complaints, and final prediction model (n = 490). [file 12888_2020_2884_MOESM1_ESM.docx]

| **Table A** Unadjusted associations with SCD-plus assessed categorically via endorsement of worries about memory complaints (*N* = 491) | | | |
| --- | --- | --- | --- |
|  | **Worries about memory complaints** (binary) | | |
|  | **Univariable logistic regression models** | | |
| **Explanatory variable** | **Odds ratio**^†^ | **95% CI** | ***p*-value** |
| Age (1 year) | 1.00 | 0.95 to 1.06 | 0.943 |
| Female (vs male) | 1.08 | 0.65 to 1.78 | 0.775 |
| Education (1 year) | 1.01 | 0.93 to 1.08 | 0.886 |
| Repetitive negative thinking (1 *SD*)^§^ | 2.18 | 1.68 to 2.82 | <0.001 |
| Rumination (1 *SD*) | 1.44 | 1.14 to 1.81 | 0.002 |
| Worry (1 *SD*) | 1.78 | 1.40 to 2.27 | <0.001 |
| Purpose in life (1 *SD*) | 0.60 | 0.48 to 0.76 | <0.001 |
| Openness to experience (1 *SD*) | 0.94 | 0.74 to 1.20 | 0.636 |
| Conscientiousness (1 *SD*) | 0.71 | 0.56 to 0.90 | 0.005 |
| Extraversion (1 *SD*) | 0.69 | 0.54 to 0.89 | 0.004 |
| Agreeableness (1 *SD*) | 0.84 | 0.66 to 1.07 | 0.151 |
| Neuroticism (1 *SD*) | 1.62 | 1.27 to 2.06 | <0.001 |
| Regular meditation practice (vs no practice) | 1.43 | 0.66 to 3.11 | 0.368 |
| Abbreviations: *SCD*, subjective cognitive decline; *SD*, standard deviation; CI, confidence interval.  ^†^In the univariable logistic regression models, for binary explanatory variables (sex, regular meditation practice), the estimate describes the odds of worries about memory complaints in one group relative to the reference category (indicated in parentheses). For continuous explanatory variables, the estimate reflects the expected increase in the odds of worries about memory complaints for a one unit increase in the explanatory variable.  ^§^*n* = 490 | | | |

| **Table B** Adjusted associations with SCD-plus assessed categorically via endorsement of worries about memory complaints, and final prediction model (*n* = 490) | | | |  |  |  |  |
| --- | --- | --- | --- | --- | --- | --- | --- |
|  | **Worries about memory complaints** (binary) | | |  |  |  |  |
|  | **Adjusted multivariable logistic regression model**^†^ | | |  |  |  |  |
| **Explanatory variables** | **Odds ratio**^‡^ | **95% CI** | ***p*-value** |  |  |  |  |
| Age (1 year) | 1.02 | 0.96 to 1.08 | 0.563 |  |  |  |  |
| Female (vs male) | 1.13 | 0.65 to 1.98 | 0.660 |  |  |  |  |
| Education (1 year) | 1.03 | 0.94 to 1.11 | 0.552 |  |  |  |  |
| Repetitive negative thinking (1 *SD*) | 2.05 | 1.35 to 3.10 | 0.001 |  |  |  |  |
| Rumination (1 *SD*) | 0.89 | 0.64 to 1.22 | 0.456 |  |  |  |  |
| Worry (1 *SD*) | 1.08 | 0.67 to 1.73 | 0.752 |  |  |  |  |
| Purpose in life (1 *SD*) | 0.91 | 0.67 to 1.23 | 0.550 |  |  |  |  |
| Conscientiousness (1 *SD*) | 0.84 | 0.64 to 1.10 | 0.206 |  |  |  |  |
| Extraversion (1 *SD*) | 0.87 | 0.66 to 1.15 | 0.329 |  |  |  |  |
| Neuroticism (1 *SD*) | 0.95 | 0.65 to 1.39 | 0.795 |  |  |  |  |
|  | **Final prediction model**^§^ | | |  |  |  |  |
| **Explanatory variables** | **Odds ratio**^‡^ | **95% CI** | ***p*-value** |  |  |  |  |
| Age (1 year) | 1.02 | 0.96 to 1.08 | 0.603 |  |  |  |  |
| Female (vs. male) | 1.02 | 0.60 to 1.74 | 0.929 |  |  |  |  |
| Education (1 year) | 1.02 | 0.95 to 1.11 | 0.544 |  |  |  |  |
| Repetitive negative thinking (1 *SD*) | 2.20 | 1.70 to 2.86 | <0.001 |  |  |  |  |
| One participant had missing data on repetitive negative thinking and thus all models include 490 participants. Abbreviations: *SCD*, subjective cognitive decline; *SD*, standard deviation; CI, confidence interval.  ^†^The adjusted models include all explanatory variables with p<0.01 in the univariable regression models. Age, sex, and education were retained based on their well-established association with dementia.  ^‡^In the multivariable logistic regression model, for binary explanatory variables (sex), the estimate describes the odds of worries about memory complaints in one group relative to the reference category (indicated in parentheses) when controlling for all other variables in the model. For continuous explanatory variables, the estimate reflects the expected increase in the odds of worries about memory complaints for a one unit increase (indicated in parentheses) in the explanatory variable when controlling for all other variables in the model.  ^§^The final prediction models retained all predictors with p<0.01 in the adjusted models. Age, sex, and education were retained based their well-known association with dementia. | | | |  |  |  | 0.0362763 |
